# Association of autoantibodies against the M2-muscarinic receptor with perinatal outcomes in women with severe preeclampsia

**DOI:** 10.1186/1479-5876-11-285

**Published:** 2013-11-11

**Authors:** Yanfang Li, Guiling Ma, Zhiyong Zhang, Yin Yue, Yuting Yuan, Yidan Wang, Guobin Miao, Lin Zhang

**Affiliations:** 1 Gynaecology and Obstetrics Department, Capital Medical University, Beijing Chao-Yang Hospital, Beijing, China; 2 Heart Failure Center, Department of Cardiology, Capital Medical University, Beijing Chao-Yang Hospital, 8# Gong-Ti South Road, 100020, Beijing, China

**Keywords:** Pregnancy, Hypertension, Antibodies

## Abstract

**Background:**

The goal of this study was to test the hypothesis that autoantibodies against M_2_-muscarinic acetylcholine receptor (M_2_-AAB) are associated with severe preeclampsia and increased risk of adverse perinatal outcomes.

**Methods:**

We conducted a case–control study comparing 60 women with severe preeclampsia to 60 women with normal pregnancy and 60 non-pregnant controls. A peptide, corresponding to amino acid sequences of the second extracellular loops of the M_2_ receptor, was synthesized as antigen to test for the presence of autoantibodies, using an enzyme-linked immunosorbent assay. The frequency and titer of M_2_-AAB were compared in the 3 groups. The risk of adverse perinatal outcomes among women with severe preeclampsia in the presence of M_2_-AAB was estimated.

**Results:**

M_2_-AAB were positive in 31.7% (19/60) of patients with severe preeclampsia, in 10.0% (6/60) (p = 0.006) of normal pregnant women and in 8.3% (5/60) (p = 0.002) of non-pregnant controls. The presence of M_2_-AAB was associated with increased risk of adverse pregnancy complications (OR, 3.6; 95%CI, 1.0-12.6; p = 0.048), fetal growth restriction (OR, 6.8; 95% CI, 2.0-23.0; p = 0.002), fetal distress (OR, 6.7; 95% CI, 1.7-26.6; p = 0.007), low Apgar score (OR, 5.3; 95% CI, 1.4-20.7; p = 0.017), and perinatal death (OR, 4.3; 95% CI, 1.0-17.6; p = 0.044) among women with severe preeclampsia.

**Conclusions:**

This study demonstrates, for the first time, an increase in M_2_-AAB in patients with severe preeclampsia. Women with severe preeclampsia who are M_2_-AAB positive are at increased risk for neonatal mortality and morbidity. We posit that M_2_-AAB may be involved in the pathogenesis of severe preeclampsia.

## Background

Preeclampsia is a pregnancy-specific syndrome characterized by hypertension and proteinuria. It occurs in 3-5% pregnancies and leads to high maternal and fetal morbidity and mortality [1]. Research has shown that preeclampsia is a multi-systemic syndrome with complex pathophysiological changes, such as endothelial dysfunction, inflammatory response, activation of the coagulation system and metabolic changes [2]. In serious cases, termination of pregnancy is the only available option to prevent further deterioration of the fetus and mother [3]. To date, the underlying mechanisms responsible for the pathogenesis of preeclampsia remain unknown.

In recent years, evidence has accumulated suggesting that autoimmunity plays a role in the pathogenesis of preeclampsia. Numerous studies have shown that women with preeclampsia possess autoantibodies against angiotensin II type 1 receptors (AT_1_-AAB), which bind to and activate the AT_1_ receptor, thus provoking biological responses relevant to the pathogenesis of preeclampsia [4-8]. Recently, we found an obvious increase in the frequency of autoantibodies against adrenergic receptors, such as β_1_, β_2_, and α_1_, in patients with severe preeclampsia [9]. Previous studies have described the role of autoantibodies against M_2_-muscarinic receptors (M_2_-AAB) in several kinds of cardiovascular disease, such as Chagas disease, hypertensive heart disease, idiopathic dilated cardiomyopathy and atrial fibrillation [10-16].

Therefore, the aim of this study was to test the hypothesis M_2_-AAB are associated with severe preeclampsia and increased risk of adverse perinatal outcomes. A synthetic peptide, corresponding to the amino acid sequence of the second extracellular loop of the human M_2_ receptor, was used to test sera from patients with severe preeclampsia, normal pregnant women, and non-pregnant controls. We compared the frequency of M_2_-AAB among the three groups. The relationship between M_2_-AAB and perinatal mortality and morbidity, was also investigated.

## Methods

### Study subjects

This was a case–control study. Patients that were admitted to Beijing Chao-Yang Hospital were managed by the obstetrics faculty of Capital Medical University. A total of 60 consecutive women diagnosed with severe preeclampsia based on the criteria set by the American College of Obstetricians and Gynecologists were recruited [17]. The criteria include increased blood pressure (≥ 160 mmHg systolic or ≥ 110 mmHg diastolic on two occasions taken at least 6 hours apart after 20 weeks of gestation) in women with previously normal blood pressure or proteinuria of ≥ 5 g over 24 h. We then randomly selected 60 age-matched, pregnant women and 60 age-matched non-pregnant women who were apparently healthy and had no hypertension or proteinuria. None of the control subjects had suffered preeclampsia previously. Exclusion criteria for all three groups included diabetes mellitus, vasculitis, renal disease and autoimmune disease. Blood samples were collected from the antecubital vein, upon recruitment into the study, using tubes containing EDTA. Samples were centrifuged at 2000 × g for 10 minutes, at 4°C, within 2 h of collection. Serum samples were stored at −80°C until analyzed. We were able to collect blood samples from ten of the sixty patients with severe preeclampsia at the end of puerperium, without a scheduled follow-up. Placenta was collected from study subjects and weighed. Clinical data from mothers and infants/neonates was also collected. The research protocol was conducted in accordance with the guidelines of the World Medical Association’s Declaration of Helsinki and was performed following approval from the Medical Ethics Committee of Capital Medical University, Beijing Chao-Yang Hospital. All women that were included in the study were in the prepartum or early intrapartum period of pregnancy and all provided written informed consent before inclusion in the study.

In this study, low birth weight was defined as birth weight less than 2500 g. A gestational age of less than 37 weeks was considered to be preterm. Fetal growth restriction was operationally defined as sonographic estimated fetal weight below the 10th percentile for the gestational age. Evidence of fetal distress was considered to be a fetal heart rate of more than 160 bpm or less than 110 bpm, evaluated by electronic fetal monitoring, or the third degree of meconium-stained amniotic fluid.

### Materials

Peptide corresponding to the amino acid sequence of the second extracellular loop of the human M_2_ receptor (residues 168 to 193, V-R-T-V-E-D-G-E-C-Y-I-Q-F-F-S-N-A-A-V-T-F-G-T-A-I-A) was synthesized by Genomed (Genomed Synthesis, Inc., San Francisco, CA, U.S.) [18-20]. The purity of the peptide, as determined by HPLC using a Vydac C-18 column, was 95.6%. The molecular weight of the peptide was analyzed by mass spectrometry. A Nunc microtiter plate was purchased from Maxisorb, Kastrup, Denmark. Tween-20, thimerosal, and ABTS were obtained from Sigma, St. Louis, MO, USA. Fetal bovine serum, biotinylated goat anti-human IgG (H + L), and horseradish peroxidase-streptavidin were purchased from Zhongshan Golden Bridge Biotech, Beijing, China. The microplate reader was purchased from Molecular Devices Corp, Menlo Park, CA.

### ELISA protocol

Samples were classified as positive or negative based on the presence or absence of M_2_-AAB. An ELISA protocol, previously described by Fu et al. [20], was used for screening. Briefly, a microtiter plate was coated with 50 μL of peptide (5 mg/L) in 100 mmol Na_2_CO_3_ solution (pH = 11) and stored overnight at 4°C. The wells were saturated with PMT (1 × PBS, 1 mL/L Tween-20, and 0.1 g/L thimerosal (PBS-T) supplemented with 100 mL/L fetal bovine serum) and incubated for 1 hour at 37°C. Then 50 μL of serum was diluted from 1:20 to 1:160, positive and negative controls were added and the wells were again incubated for 1 hour at 37 °C. After washing three times with PBS-T, affinity-purified biotinylated goat anti-human IgG (H + L) (1:500 dilution in PMT) was added and the wells were incubated for 1 hour at 37°C. After another round of washing, the bound biotinylated antibody was detected by incubating the microtiter plate for 1 hour at 37°C with horseradish peroxidase-streptavidin (1:500 dilution in PMT). After washing an additional three times with PBS, 2.5 mmol/L H_2_O_2_ was added followed by 2 mmol/L ABTS in citrate buffer solution. After 20 minutes, absorbance (*A*) was measured at 405 nm in a microplate reader. The sensitivity and specificity of the ELISA assay, for sample sera and positive and negative serum, were measured by the corresponding curves. Several recently detected samples were combined for the positive and negative sera. All samples were tested twice to verify the reliability of the result. The intra-assay and inter-assay coefficient of variation was less than 5 %. The detection range of absorbance was up to 2.5. Further dilution was done when the absorbance was over the upper limit.

### Data analysis

Quantitative data are expressed as the mean ± SD. Positivity was defined as a ratio of (sample *A -* blank *A*)/(negative control *A* - blank *A*) ≥ 2.1. Antibody titer was reported as geometric mean. Continuous variables that were not normally distributed were log-transformed to obtain normality for testing, and geometric means were presented. One-way ANOVA test was used to determine significant differences between groups. The association between the presence of M_2_-AAB and categorical outcomes among women with severe preeclampsia was estimated by calculating unadjusted odds ratios. Adjusted analysis was not performed due to the small sample size. Data were analyzed using SPSS 16.0 (SPSS, Chicago, Illinois, USA). P < 0.05 was considered statistically significant.

## Results

A total of 180 women were included in the study. Of these, 60 were in the severe preeclampsia group, 60 were in the normal pregnant group and 60 were in the non-pregnant control group. Study subjects were enrolled between May 2011 and November 2012. Clinical characteristics of the women in the three study groups are shown in Table 1.

**Table 1:** Clinical characteristics of women from three groups in the present study

	**Non-pregnant**	**Normal pregnant**	**Severe preeclampsia**
	**(n = 60)**	**(n = 60)**	**(n = 60)**
Age (years)	30.4 ± 3.9	29.0 ± 0.6	29.5 ± 4.7
Gestational age (weeks)	NA	38.6 ± 0.3	33.1 ± 4.6*
Systolic blood pressure (mmHg)	118.7 ± 6.8	115.5 ± 1.6	168.0 ± 15.7*
Diastolic blood pressure (mmHg)	74.7 ± 6.3	73.9 ± 1.4	109.6 ± 12.4*
Urinary protein (mg/24 h)	Nd†	Nd†	6448.1 ± 2814.6

Data are mean ± SD. Student’s unpaired two-tailed t-test was used to compare the non-pregnant to the normal pregnant group and the normal pregnant group to the severe preeclampsia group. Significant differences are indicated by * (p < 0.001).

Nd: not determined; NA: not applicable.

†: Urine protein of normal pregnant and non-pregnant women was within normal ranges and not routinely recorded.

### Maternal clinical characteristics

Headache was the main complaint in the severe preeclampsia group. Blurred vision, epigastric pain, and oliguria were also common complaints.

The maternal hospital stay was significantly longer for women in the severe preeclampsia group compared with those in the normal pregnant group (9.1 ± 5.4 days versus 4.2 ± 2.3 days, p < 0.001). The frequency of pregnancy complications, including oligohydramnios (6/60), placental abruption (5/60), placenta remnants (7/60), postpartum hemorrhage (4/60), retinal edema (2/60), preretinal hemorrhage (4/60) and hypertensive retinopathy (8/60), was significantly higher among those in the severe preeclampsia group than in the normal pregnant group (36/60 versus 0/60, p < 0.001).

### Perinatal clinical characteristics

Fetal ultrasound examination showed significant elevations in pulse index, resistance index and the S/D value of the umbilical artery. S/D value refers to the ratio of the peak systolic and diastolic velocity of the fetal umbilical artery and is indicative of the placenta-fetal blood flow resistance.

A total of 41.7% (25/60) of fetuses in the severe preeclampsia group suffered from fetal growth restriction and 20.0% (12/60) suffered from fetal distress; both of which were significantly higher compared with fetuses in the normal pregnant group (p < 0.001 for both). The proportion of preterm births and low birth weight was significantly higher in the severe preeclampsia group compared with the normal pregnant group (76.7% versus 10.0% and 75.0% versus 6.7%, p < 0.001, respectively). The proportion of perinatal deaths was also higher in the severe preeclampsia group than in the normal pregnant group (16.7% versus 0%, p < 0.001) (Table 2).

**Table 2:** Perinatal complications

**Complications**	**Severe preeclampsia n = 60 (%)**	**Normal pregnant n = 60 (%)**	**P value**
Fetal growth restriction	25(41.7)	1(1.7)	<0.001†
Fetal distress	12(20.0)	2(3.3)	0.008*
Low birth weight	45(75.0)	4(6.7)	<0.001†
<1500 g	15(25.0)	0	<0.001†
<1000 g	5(8.3)	0	0.068
Preterm	46(76.7)	6(10.0)	<0.001†
Neonatal asphyxia	12(20.0)	0	<0.001†
Mild	8(13.3)	0	0.010*
Severe	4(6.7)	0	0.127
Perinatal death	10(16.7)	0	<0.001†
Intrauterine death	6(10.0)	0	0.036*
Neonatal death	4(6.7)	0	0.127

*P < 0.05; †p < 0.001.

Birth weight in the severe preeclampsia group was significantly lower than in the normal pregnant group (2142.1 ± 786.8 g versus 3279.1 ± 359.4 g, p < 0.001). Similarly, placental weight was also lower in the severe preeclampsia group compared with the normal pregnant group (517.9 ± 237.6 g versus 650.6 ± 120.6 g, p < 0.001).

Neonatal Apgar score (appearance of skin color, pulse, grimace, activity, and respiration) was used to classify newborn infants [21]. The Apgar scores were significantly lower in infants from the severe preeclampsia group compared with infants from the normal pregnant group at one minute (7.1 ± 1.8 versus 9.5 ± 0.5, p < 0.001), five minutes (8.0 ± 1.3 versus 9.8 ± 0.2, p < 0.001), and ten minutes (8.3 ± 1.4 versus 10.0 ± 0, p < 0.001). Low Apgar score was defined, according to the Apgar score at one minute; severe was considered to be between 0 and 3 and mild was considered to be between 4 and 7. The incidence of low Apgar score was significantly higher in the severe preeclampsia group compared with the normal pregnant group (20.0% versus 0%, respectively; p < 0.001).

Of the 60 neonates in the severe preeclampsia group, 16.7% (10/60) died, 21.7% (13/60) were transferred to the BaYi Children’s Hospital, 41.7% (25/60) were transferred to the pediatric department of our hospital, and 20.0% (12/60) required no further treatment.

### The sensitivity and specificity of the ELISA assay

**Figure 1:**
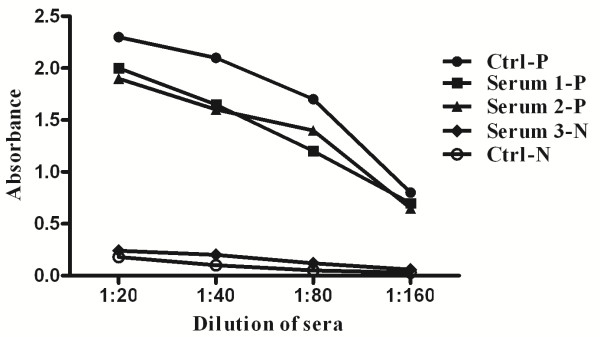
**The sensitivity and specificity of the ELISA assay.** The curve of positive patients (3 randomly selected samples) was in good correspondence with the curve for the positive control blood sample, and the similar result was found in negative samples. There was a great difference in response between patients positive and those negative to the M_2_-AAB.

Results of samples from most of the patients were stable until the titer serum was diluted to 1:160. Although the absorbance decreased when the sera were diluted, there was a significant difference between the positive and negative samples throughout. The sensitivity and specificity of the assay to the M_2_-AAB are shown in Figure 1. The curve for positive patient samples (3 randomly selected samples) corresponded well with the curve for the positive control sample. Results were similar for negative samples. There was a large difference in response between samples from patients that were positive for M_2_-AAB and those that were negative for M_2_-AAB. Based on these curves, we concluded that the ELISA had good sensitivity and specificity.

### ELISA result

**Figure 2:**
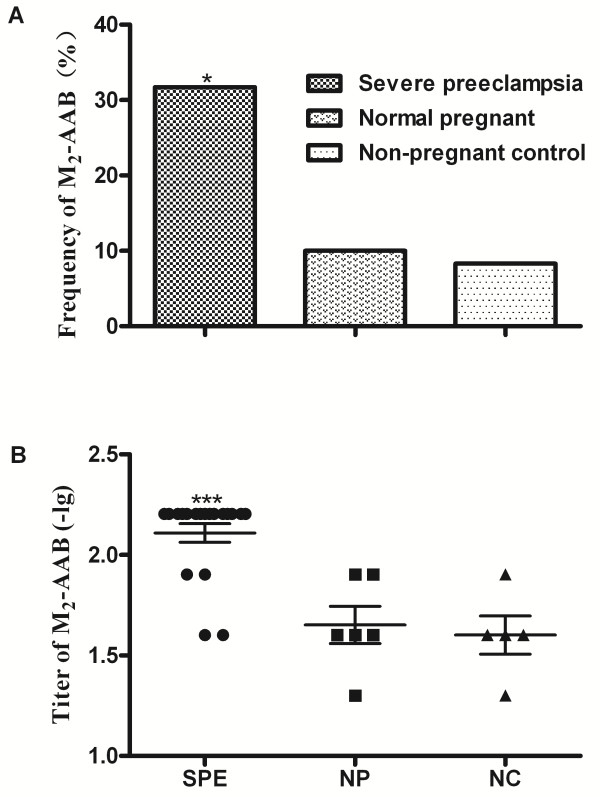
**Frequencies and titers of autoantibodies among the three groups.** Panel **A**: Frequencies of M_2_-AAB was significantly higher in women with severe preeclampsia than in the normal pregnant women and non-pregnant controls. Panel **B**: Geometric mean titers of M_2_-AAB was significantly higher in women with severe preeclampsia than in the normal pregnant women and non-pregnant controls. *p < 0.05: women with severe preeclampsia compared to normal pregnant women and non-pregnant controls; ***p < 0.001: women with severe preeclampsia compared to normal pregnant women and non-pregnant controls. M_2_-AAB: autoantibodies against M_2_-muscarinic receptors; SPE: severe preeclampsia; NP: normal pregnant; NC: non-pregnant control.

A total of 31.7% (19/60) of the severe preeclampsia group, 10.0% (6/60) (p = 0.006) of the normal pregnant group, and 8.3% (5/60) (p = 0.002) of non-pregnant controls were sera positive for M_2_-AAB. The geometric mean titer of the M_2_-AAB was significantly higher in the severe preeclampsia group (1:128) compared to the normal pregnant group (1:44) and to the non-pregnant controls (1:40) (p < 0.001 for both) (Figure 2).

### The association of M_2_-AAB and clinical outcomes

Unadjusted odds ratios were used to estimate the association of M_2_-AAB with pregnancy complications, fetal distress, preterm birth, neonatal asphyxia, and perinatal death among women with severe preeclampsia. Positivity for M_2_-AAB was associated with pregnancy complications (OR, 3.6; 95%CI, 1.0-12.6; p = 0.048), fetal growth restriction (OR, 6.8; 95% CI, 2.0-23.0; p = 0.002), fetal distress (OR, 6.7; 95% CI, 1.7-26.6; p = 0.007), low Apgar score (OR, 5.3; 95% CI, 1.4-20.7; p = 0.017), and perinatal death (OR, 4.3; 95% CI, 1.0-17.6; p = 0.044).

## Discussion

### Main findings

In this study we demonstrated, for the first time, that positivity for M_2_-AAB is closely associated with severe preeclampsia. The frequencies and titers of M_2_-AAB were significantly higher in the severe preeclampsia group, when compared to normal pregnant women and non-pregnant healthy controls. The presence of M_2_-AAB in women with severe preeclampsia was associated with both adverse maternal and perinatal clinical outcomes, including pregnancy complications, fetal distress, fetal growth restriction, low Apgar score and perinatal death.

### Immune mechanisms in preeclampsia

The pathogenesis of preeclampsia remains obscure, but is likely multifactorial and involves abnormal placentation, reduced placental perfusion, endothelial cell dysfunction, and systemic vasospasm [22]. An immune mechanism has long been postulated as playing a role in the pathogenesis of preeclampsia. Immune maladaptation may impair invasion of spiral arteries by endovascular cytotrophoblast cells [23]. Studies have suggested that repeated exposure to sperm from a particular male partner prior to pregnancy promotes immune tolerance and reduces the risk of defective trophoblast invasion [24]. Autoantibodies, such as anticardiolipin and anti-β2-glycoprotein-1 antibody, have been detected in preeclampsia patients [25]. Since the first report was published that described the presence of AT_1_-AAB in preeclampsia patients [4], researchers have gained a greater understanding of the pathogenic mechanisms underlying preeclampsia, which implicate the immune system. Recently we found an obvious increase in the frequency of autoantibodies against adrenergic receptors, such as β_1_, β_2_, and α_1_, in patients with severe preeclampsia with obscure mechanisms [9].

### Autoantibodies and preeclampsia

While our results need to be confirmed by larger studies, there are biologically plausible mechanisms by which M_2_-AAB may lead to severe preeclampsia. The M_2_ receptor is primarily expressed in the heart (in human and other mammalian species), and its activation results in negative chronotropic and inotropic effects by inhibiting adenylyl cyclase, decreasing intracellular cAMP, and reducing L-type Ca^2+^ currents. Previous studies from our group and others have demonstrated that M_2_-AAB display “agonistic activity” against their target receptors resulting in myocardial injury and cardiac dysfunction.

Studies have shown that the risk of long-term sequelae, such as chronic hypertension, ischemic heart disease, stroke, and venous thromboembolism are significantly increased in women with preeclampsia [26,27]. In this study, we were able to collect blood samples from ten of the sixty patients with severe preeclampsia at the end of puerperium, without a scheduled follow-up. Three of the ten samples were positive for M_2_-AAB at titers similar to the levels at the time of recruitment. This is similar to what has been observed for autoantibodies against adrenergic receptors [9]. We infer that the presence of autoantibodies might be correlated to a high risk for cardiovascular sequelae, however this hypothesis needs further exploration.

Frequencies and titers of M_2_-AAB were significantly higher in the severe preeclampsia group than in the normal pregnant women and non-pregnant control groups. Therefore, we hypothesize that there may be a relationship between the presence of M_2_-AAB and the development of severe preeclampsia. Alternatively, it is plausible that severe preeclampsia triggers the production of M_2_-AAB. Further studies are needed to clarify the association between M_2_-AAB and the development of severe preeclampsia.

### Limitations

There are limitations that should be considered when interpreting our results. First, this case–control study, like all case–control studies, there is always that possibility of selection bias. We included two groups of controls who were age-matched to the cases and were randomly selected from the sample population. This would minimize selection bias. Second, our analyses were unadjusted for potential confounders. While this is unlikely, it is possible that the associations observe could be the result of an unknown confounder. Third, while the association between M_2_-AAB and severe preeclampsia is biologically plausible, we are careful to point out that association is not necessarily causality. Further studies will be needed to elucidate a causal role of M_2_-AAB in severe preeclampsia. However, the association between M_2_-AAB and adverse perinatal outcomes among women with severe preeclampsia is suggestive of a causal role. Fourth, as we didn’t include a group of mild preeclampsia patients, the relation between severity of preeclampsia and the antibody titer was unknown. Besides, maybe gestational age affects the antibody titer more than maternal age does. We collect blood samples of patients that were admitted to Beijing Chao-Yang Hospital during prepartum period and the gestational age was significant increased in the normal pregnant women. Finally, only serum M_2_-AAB was detected. Further studies that examine the biological activity of M_2_-AAB, as well as M_2_ receptors in the placenta and umbilical vessels, are needed.

## Conclusion

This study demonstrates the prevalence of M_2_-AAB in a cohort of women with severe preeclampsia. Risks of both maternal and perinatal complications are significantly increased when M_2_-AAB is present. M_2_-AAB may participate in the pathogenesis of severe preeclampsia and have clinical value for predicting complications.

## Competing interests

The authors declare that they have no competing interests.

## Authors’ contributions

YFL, GLM, ZYZ and YY carried out the case, blood sample and clinical data collection. GLM and YDW carried out the immunoassay. GLM and GBM performed the analysis and interpretation of data. GLM and YTY were involved in drafting part of the manuscript. LZ contributed the whole study and participated in the design and coordination of this project as well as manuscript writing. All authors reviewed and approved the final manuscript.
